# Improved detection of in-transit metastases of malignant melanoma with BSREM reconstruction in digital [^18^F]FDG PET/CT

**DOI:** 10.1007/s00330-021-07852-7

**Published:** 2021-03-25

**Authors:** Virginia Liberini, Michael Messerli, Lars Husmann, Ken Kudura, Hannes Grünig, Alexander Maurer, Stephan Skawran, Erika Orita, Daniele A. Pizzuto, Désirée Deandreis, Reinhard Dummer, Joanna Mangana, Daniela Mihic-Probst, Niels Rupp, Martin W. Huellner

**Affiliations:** 1 Department of Nuclear Medicine, University Hospital Zürich, University of Zürich, Rämistrasse 100, CH-8091 Zürich, Switzerland; 2 Department of Nuclear Medicine, Città della Salute e della Scienza di Torino, University of Turin, Turin, Italy; 3 Department of Radiology, Nippon Medical School, 1-1-5 Sendagi, Bunkyo-ku, Tokyo, 113-8603 Japan; 4 Nuclear Medicine Unit, Fondazione Policlinico Universitario A. Gemelli IRCCS, 00168 Rome, Italy; 5 Department of Dermatology, University Hospital Zurich, University of Zurich, Zurich, Switzerland; 6 Department of Pathology and Molecular Pathology, University Hospital Zurich, University of Zurich, Zurich, Switzerland

**Keywords:** Fluorodeoxyglucose F18, Positron Emission Tomography / Computed Tomography, Melanoma, Algorithms, Skin neoplasms

## Abstract

**Objectives:**

To compare block sequential regularized expectation maximization (BSREM) and ordered subset expectation maximization (OSEM) for the detection of in-transit metastasis (ITM) of malignant melanoma in digital [^18^F]FDG PET/CT.

**Methods:**

We retrospectively analyzed a cohort of 100 [^18^F]FDG PET/CT scans of melanoma patients with ITM, performed between May 2017 and January 2020. PET images were reconstructed with both OSEM and BSREM algorithms. SUVmax, target-to-background ratio (TBR), and metabolic tumor volume (MTV) were recorded for each ITM. Differences in PET parameters were analyzed with the Wilcoxon signed-rank test. Differences in image quality for different reconstructions were tested using the Man-Whitney *U* test.

**Results:**

BSREM reconstruction led to the detection of 287 ITM (39% more than OSEM). PET parameters of ITM were significantly different between BSREM and OSEM reconstructions (*p* < 0.001). SUVmax and TBR were higher (76.5% and 77.7%, respectively) and MTV lower (49.5%) on BSREM. ITM missed with OSEM had significantly lower SUVmax (mean 2.03 vs. 3.84) and TBR (mean 1.18 vs. 2.22) and higher MTV (mean 2.92 vs. 1.01) on OSEM compared to BSREM (all *p* < 0.001).

**Conclusions:**

BSREM detects significantly more ITM than OSEM, owing to higher SUVmax, higher TBR, and less blurring. BSREM is particularly helpful in small and less avid lesions, which are more often missed with OSEM.

**Key Points:**

*• In melanoma patients, [* ^*18*^ *F]FDG PET/CT helps to detect in-transit metastases (ITM), and their detection is improved by using BSREM instead of OSEM reconstruction.*

*• BSREM is particularly useful in small lesions.*

**Supplementary Information:**

The online version contains supplementary material available at 10.1007/s00330-021-07852-7.

## Introduction

Cutaneous malignant melanoma (CMM) is the 5^th^ most common cancer in men and the 6^th^ most common cancer in women worldwide [1, 2]. The incidence of CMM increased in the last 40 years, partly attributable to improved screening programs, with approximately 287,700 new annual cases globally [3].

Cutaneous and subcutaneous melanoma metastases are very frequent and include microsatellite, satellite, and in-transit metastases (ITMs). With the 8^th^ edition of the American Joint Committee on Cancer (AJCC), these three different entities were merged into the single subcategory “c” of the N classification (N1c, N2c, and N3c) [4]. The association of such metastases with poor prognosis was demonstrated by several studies [5–9]. In particular, ITMs occur in 2–10% of melanoma patients and are frequently associated with the development of nodal and/or systemic metastases [10], even in sentinel node-negative patients [11]. In 2015, Beasley et al [12] have reported a 5-year survival rate of 59% in patients without regional nodal disease compared to 19% for those with nodal disease (including ITM).

To reduce melanoma-related mortality and distant metastasis development, an earlier detection of ITM could be helpful, although no such data exists currently. Moreover, ITM can be treated both with novel systemic agents (as immune checkpoint inhibitors and mitogen-activated protein kinase pathway inhibitors) and with locoregional interventions (as surgery, electrochemotherapy, isolated limb infusion or perfusion, and oncolytic viral therapy) [13–18].

Typically, ITMs are detected during clinical examination of patients or by ultrasound (US) using high-frequency (HF) probes, which is time-consuming, operator-dependent, and limited in terms of tissue depth and small-sized lesions. 2-Deoxy-2-[18F]fluoro-D-glucose positron emission tomography/computed tomography ([^18^F]FDG PET/CT) is typically used for the staging and restaging of high-risk melanoma patients, mainly for the detection of lymph node metastases and distant metastases. With the advent of digital PET and novel iterative reconstruction techniques, the detectability of small-sized [19] and faintly [^18^F]FDG-avid lesions has improved considerably [20, 21]. Hence, digital PET/CT may play a relevant role in the detection of the exact number, size, and location of ITM and may subsequently impact patient management and therapy-related decisions [18, 22–24]. The aim of our study was to assess the value of [^18^F]FDG PET images reconstructed with block sequential regularized expectation maximization (BSREM) compared to the clinical standard ordered subset expectation maximization (OSEM) for ITM detection.

## Material and methods

### Patient selection

We retrospectively analyzed a cohort of 1575 consecutive examinations of patients, who underwent a clinically indicated [^18^F]FDG PET/CT scan on a digital scanner for the staging/restaging of malignant melanoma at the University Hospital of Zurich between May 2017 and January 2020. Only patients with documented willingness to the use of their medical data for research were included (423 examinations excluded) in this retrospective, observational study. Our study was approved by the local ethics committee and was conducted in compliance with ICH-GCP rules and the Declaration of Helsinki. All reports of the remaining 1152 PET/CT scans were reviewed for reported ITM presence. In each reported case, the ITM presence on imaging was verified by one doubly board-certified radiologist/nuclear medicine physician with 12 years of experience in oncological hybrid imaging (M.H.).

Hence, eligible patients matched all the following inclusion criteria: (a) histologically proven melanoma; (b) presence of at least one in-transit metastasis described in report (based on BSREM algorithm) and verified by the above-mentioned reader; (c) PET/CT scan acquired on a digital scanner with silicon photomultiplier (SiPM) technology; (f) availability of both OSEM and BSREM reconstructions. The final study cohort consisted of a total of 100 examinations.

At our institution, BSREM serves as clinical standard for all oncological [^18^F]FDG PET exams carried out on digital scanners, and OSEM is reconstructed by default in order to ensure comparability with analog scanners without BSREM technology. Pathological confirmation, clinical examination including ultrasound, and outcome and/or imaging after 3–6 months served as the standard of reference for proving ITM. In 65 of the 100 PET/CT scans, at least one ITM was histologically proven. In the remaining 35 cases, the location of the lesion (between the primary site and regional nodal basin, along the lymphatic stream), clinical examination including ultrasound, and outcome and/or imaging after 3–6 months served as the standard of reference for the designation of a lesion as ITM.

### PET/CT acquisition

All included patients underwent a PET/CT scan on a digital scanner with SiPM technology (GE Discovery Molecular Insights - DMI PET/CT, GE Healthcare). The injected tracer activity was 221.53 ± 6.67 MBq of [^18^F]FDG. After an uptake time of 60 min and following CT acquisition both for attenuation correction and anatomical correlation, PET data were acquired in 3-dimensional time-of-flight (TOF) mode, covering the identical anatomical region of the CT.

PET image datasets were reconstructed with different standardized settings (all with a 256 × 256 pixel matrix):

1. OSEM: 3 iterations, 16 subsets, FWHMI of 6.3 mm, 1:4 *Z*-axis filter, and 6.4-mm Gaussian filter with both time-of-flight (TOF) and point spread function (PSF) modeling (OSEM_PSF_; VUE Point FX with SharpIR, GE Healthcare).
2. BSREM (Q.Clear, GE Healthcare) with both TOF and PSF and a β-value of 450 (BSREM_450_) which represents the institutional standard [25–28].

### Quantitative imaging analysis

Quantitative analysis was performed by two readers, blinded to clinical data. Readers were provided with de-identified images reconstructed with OSEM and BSREM, in random patient and reconstruction order. The task of in-transit metastasis detection was assigned to the readers, and readers recorded the slice position and SUVmax of all lesions detected. PET images were segmented using a dedicated workstation (GE Healthcare). The following indices were recorded for each lesion: location, metabolic tumor volume (MTV), maximum standardized uptake value (SUVmax), and mean standardized uptake value (SUVmean).

PET parameters were measured on PET images using a volume of interest (VOI) including the whole lesion volume, outlined with a 3D semi-automatic contouring tool, and applying a threshold set at 42% of the SUVmax. Target-to-background ratios (TBR), defined as the ITM SUVmax corrected for physiological blood pool SUVmean, were also calculated [29]. All quantitative image analyses were performed on both OSEM and BSREM reconstructions, using cloned VOIs for both reconstructions.

### Statistical analysis

Categorical variables are expressed as proportions, and continuous variables are presented as mean ± standard deviation (SD) or median (range), depending on the distribution of values. ﻿We assessed the number of ITM detected with either reconstruction algorithm. Moreover, we assessed the frequency of PET parameter changes (SUVmax, TBR, and MTV) comparing BSREM with OSEM. Based on PET Response Criteria in Solid Tumors (PERCIST), we considered a change in PET parameters of ± 30% (BSREM vs. OSEM) as clinically relevant [30]. The Wilcoxon signed-rank test was used to test for differences in lesional PET parameters among both reconstructions. Differences in image quality among reconstructions were tested using the Mann-Whitney *U* test and the Pearson test, with linear regression used to calculate the correlation coefficients. Statistical significance was considered for *p* < 0.05. Statistical analyses were performed using SPSS version 26.0 (IBM) [31].

## Results

### Patient and tumor characteristics

Patient and tumor characteristics are listed in Table 1 and supplemental table S1. Eighty-eight percent of the scans were performed for restaging, and 12% for initial staging purposes. Before PET/CT, all patients had already undergone surgery (100%; primary tumor and/or lymph node surgery) and several other treatments, such as chemotherapy (4%), small molecule targeted therapy (15%), immunotherapy (47%), radiotherapy (21%), TVEC (10, and other therapies (19%).

**Table 1:** Patient and primary tumor characteristics

Patient characteristics	
PET/CT scan, *n* (%)	
Staging	12 (12.0)
Restaging	88 (88.0)
Gender, *n* (%)	
Male	60 (60.0)
Female	40 (40.0)
Age (years), median (range)	64.50 (21–91)
Activity injected (MBq), median (range)	229.50 (97–330)
Uptake time (min), median (range)	60.00 (43–92)
Blood glucose level (mmol/L), median (range)	5.20 (4.2–7.8)
Weight (kg), median (range)	77.50 (50–114)
Height (cm), median (range)	172.00 (147–195)
BMI (kg/m^2^), median (range)	26.75 (19.7–38.8)
Primary melanoma characteristics	
Type, *n* (%)	
Superficial spreading melanoma (SSM)	22 (22.0)
Lentigo malignant melanoma (LMM)	2 (2.0)
Acral lentiginous melanoma (ALM)	9 (9.0)
Nodular melanoma (NM) Unknown	41 (41.0) 19 (19.0)
Location, *n* (%)	
Head and neck	10 (10.0)
Torso	23 (23.0)
Arms	8 (8.0)
Legs	51 (51.0)
Unknown	8 (8.0)
Clark level, *n* (%)	
II	2 (2.0)
III	10 (10.0)
IV	37 (37.0)
V	11 (11.0)
Unknown	40 (40.0)
Breslow, mean ± SD (range)	2.8 ± 2.5 (0.6–20.0)
Breslow, n (%)	
*< 1.0 mm*	3 (3.0)
*1.0–2.0 mm*	31 (31.0)
*2.1–4.0 mm*	32 (32.0)
*> 4.0 mm*	23 (23.0)
*Unknown*	11 (11.0)
Ulceration, *n* (%)	
Yes	54 (54.0)
No	28 (28.0)
Unknown	18 (18.0)
BRAF mutation, *n* (%)	
Yes	37 (37.0)
No	35 (35.0)
Unknown	28 (28.0)

*Note*: *BMI*, body mass index; *BRAF*, v-Raf murine sarcoma viral oncogene homolog B; *MBq*, megabecquerel; *PET*, positron emission tomography

In our study, the mean follow-up time after the analyzed PET/CT scan was 18.2 ± 1.5 (0.0–131.0) months. Fifty-four PET/CT scans detected only ITM but no nodal metastases, 14 PET/CT scans detected ITM and lymph node metastases, and 32 PET/CT scans detected ITM and distant metastasis. According to RECIST 1.1 criteria, favorable outcome (complete response + partial response + stable disease) was higher in patients with only ITM (34/54; 34% of the entire cohort) compared to patients with ITM and lymph node metastases (10/14; 10%) and patients with ITM and distant metastases (15/32; 15%).

### Differences in PET parameters between OSEM and BSREM algorithms

Lesions suspected to represent ITM were detected by the readers in all 100 PET/CT scans included in this study. The majority of ITM (69.0%) were located in the legs, 16.4% in the torso, 8.0% in the arms, and 6.6% in the head and neck.

Readers detected a total of 295 lesions with BSREM, 214 with OSEM. Overall, 8 of the detected lesions turned out false positive (6 granulomatous inflammations, 2 lymph node metastases, all confirmed by histopathology) in 8 subjects, who underwent a restaging PET/CT scan. All of these lesions were recorded by the readers with both BSREM and OSEM. All 8 false-positive lesions were present in subjects who had also true positive lesions, i.e., ITM. Interestingly, all these false-positive lesions were also initially considered clinically to represent ITM.

Using BSREM, readers correctly detected a total of 287 ITM, of which 206 were detected also with OSEM (difference of 39%), equaling a mean per patient ITM number of 2.06 for OSEM and 2.87 for BSREM (*p* < 0.001). In 20 PET/CT scans (20% of cohort), ITM presence was detected only by BSREM, but not by OSEM. With BSREM/OSEM, the number of in-transit metastases detected per patient was 1 lesion (42% / 25% of cases, respectively), 2–5 lesions (44%/41%), and > 5 lesions (14%/14%).

**Fig. 1:**
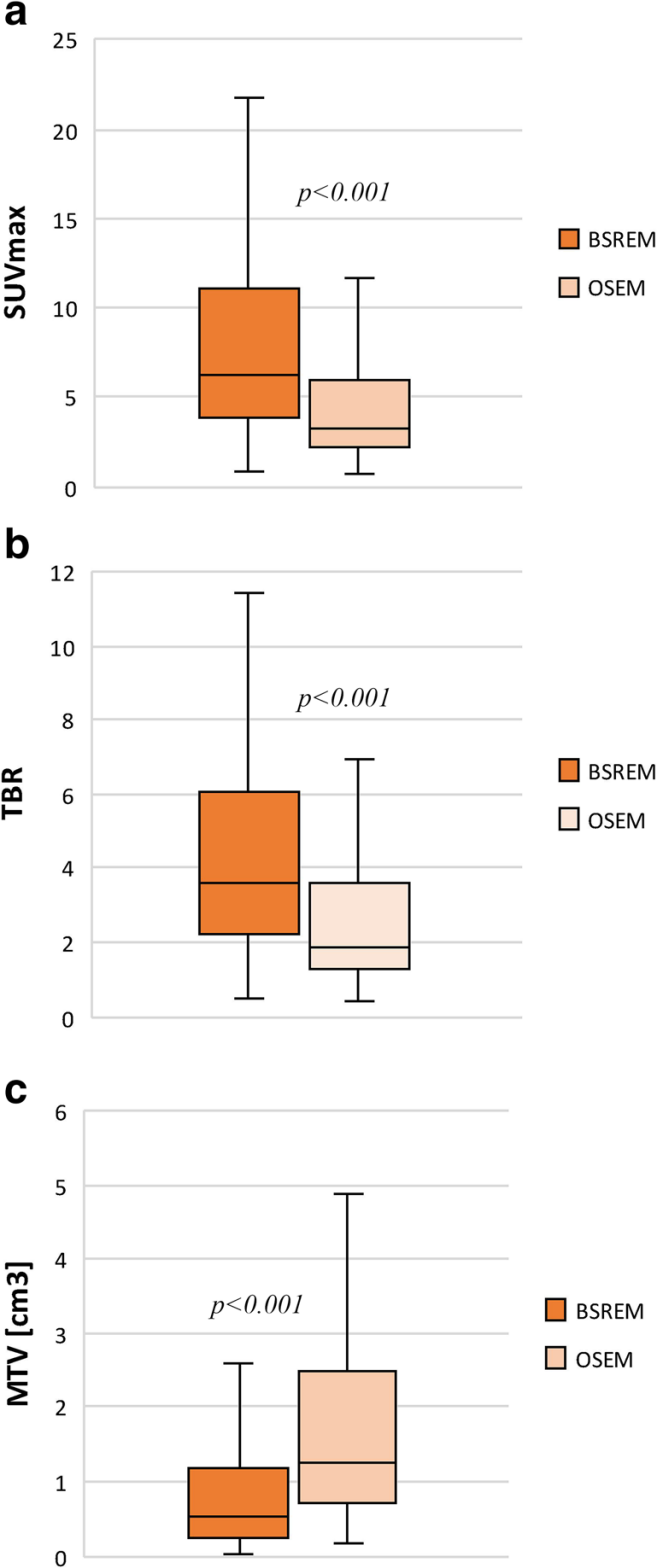
PET parameters (**a** SUVmax, **b** TBR, **c** MTV) of in-transit metastasis with OSEM and BSREM reconstruction

All PET parameters (SUVmax, TBR, MTV) were significantly different between BSREM and OSEM reconstructions (*p* < 0.001), both for the entire cohort and for several sub-groups, such as ITM detected only with BSREM (retrospectively analyzed also with OSEM) and ITM in different anatomical locations (head and neck, torso, arms, and legs respectively). In the entire cohort, there was a difference (BSREM vs. OSEM; all *p* < 0.001) in ITM SUVmax of +76.5% (mean 8.42 vs. 4.77), TBR of +77.7% (mean 4.78 vs. 2.69), and MTV of - 49.5% (mean 1.01 vs. 2.00 cm^3^). Figure 1 shows the box plots of ITM PET parameters in OSEM and BSREM, respectively.

The latter result is consistent with the fact that BSREM detects smaller lesions. As shown in Table 2, all these differences were more pronounced for ITM that were missed with OSEM reconstruction and retrospectively analyzed. Here, we observed an increase in ITM SUVmax by +89.2% from OSEM to BSREM (mean 2.03 vs. 3.84, respectively, *p < *0.001), in ITM TBR by +88.1% (mean 1.18 vs. 2.22, respectively, *p* < 0.001), and a decrease in ITM MTV by -65.4% (mean 2.92 vs. 1.01 cm^3^, respectively, *p* < 0.001).

**Table 2:** Characteristics of the in-transit metastases in OSEM and BSREM

	OSEM	BSREM	Parameter difference in BREM vs. OSEM (%)	*p* value*
In-transit metastasis (ITM) detected				
Overall ITM number, *n*	206	287 (+81)	+ 39.32%	-
Mean ITM per patient, mean ± SD; min-max	2.06 (± 2.29; 0–12)	2.87 (± 2.56; 1–12)	+ 39.32%	-
ITM detected per patient, *n*				
1 lesion	25	42 (+17)	+ 68.00%	-
2–5 lesions	41	44 (+3)	+ 7.31%	-
> 5 lesions	14	14 (+0)	+ 0.00%	-
ITM location, *n* (%)				
Head and neck	6	19 (+ 13)	+ 216.66%	-
Torso	34	47 (+ 13)	+ 38.23%	-
Arms	17	23 (+ 6)	+ 35.29%	-
Legs	149	198 (+ 48)	+ 32.21%	-
Blood pool SUVmean, mean ± SD; min-max	1.76 ± 0.24 (1.19–2.59)	1.77 ± 0.24 (1.19–2.58)	+ 0.56%	0.860
SUVmax, mean ± SD; min-max	4.77 ± 4.34 (0.68–46.32)	8.42 ± 7.39 (0.84–71.60)	+ 76.51%	< 0.001
TBR, mean ± SD; min-max	2.69 ± 2.30 (0.39–24.90)	4.78 ± 4.20 (0.48–38.28)	+ 77.69%	< 0.001
MTV (cm^3^), mean ± SD; min-max	2.00 ± 1.97 (0.18–10.01)	1.01 ± 1.33 (0.02–8.39)	−49.54%	< 0.001
In-transit metastasis (ITM) missed by OSEM reconstruction				
Overall ITM number, *n*	**-**	81	-	
ITM location, *n* (%)				
Head and neck	-	13	-	
Torso	-	13	-	
Arms	-	6	-	
Legs	-	49	-	
SUVmax, mean ± SD; min-max	2.03 ± 0.71 (0.68–4.41)	3.84 ± 1.78 (0.84–10.99)	+ 89.16%	< 0.001
TBR, mean ± SD; min-max	1.18 ± 0.47 (0.39–3.39)	2.22 ± 1.02 (0.48–5.69)	+ 88.13%	< 0.001
ITM MTV (cm^3^), mean ± SD; min-max	2.92 ± 2.32 (0.39–9.24)	1.01 ± 1.16 (0.04–6.17)	− 65.41%	< 0.001

*Note*: *ITM*, in-transit metastasis; *TBR*, target-to-background ratio

*p value was calculated with the Wilcoxon signed-rank test

**Fig. 2:**
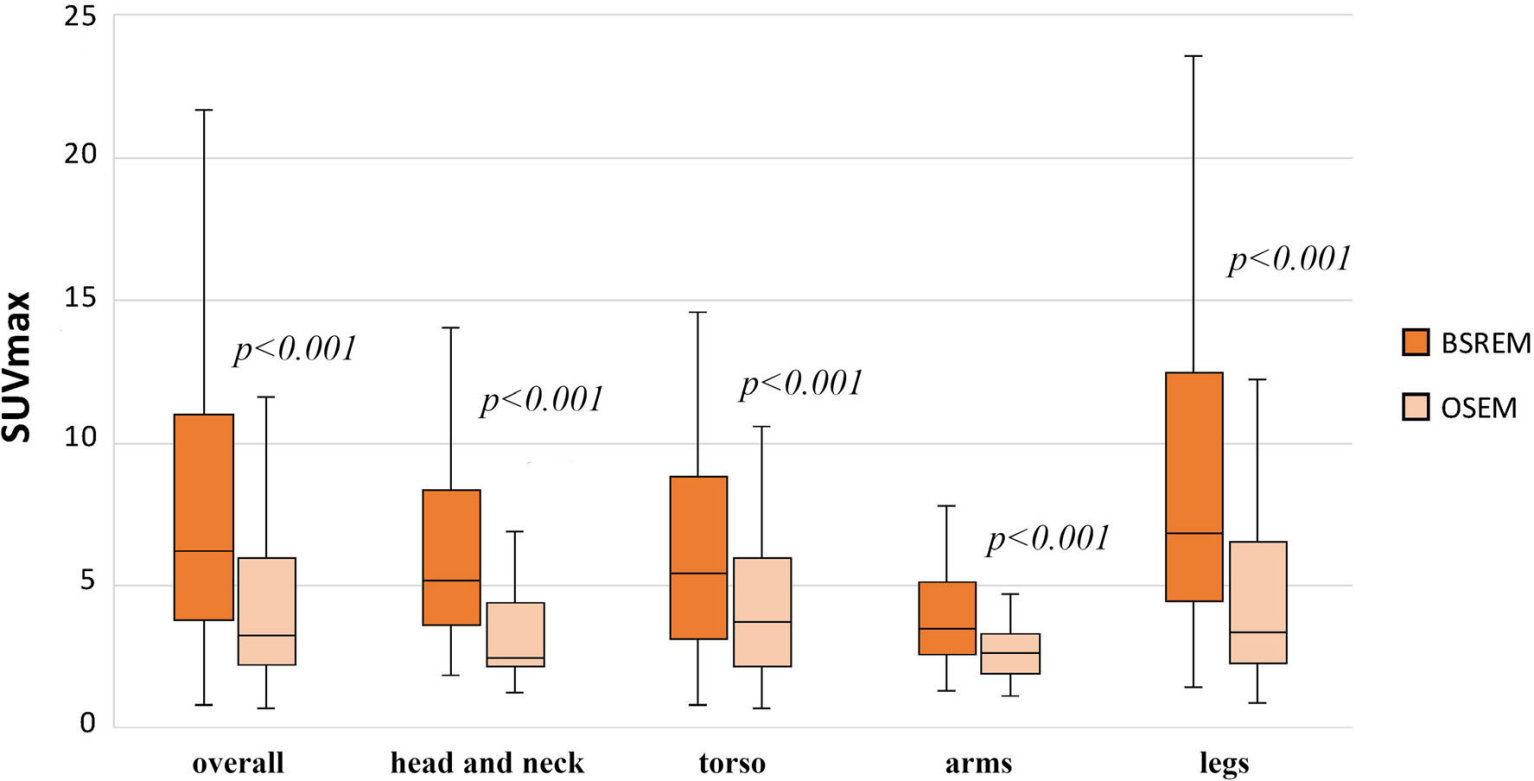
SUVmax of ITM in OSEM and BSREM reconstructions by location

**Fig. 3:**
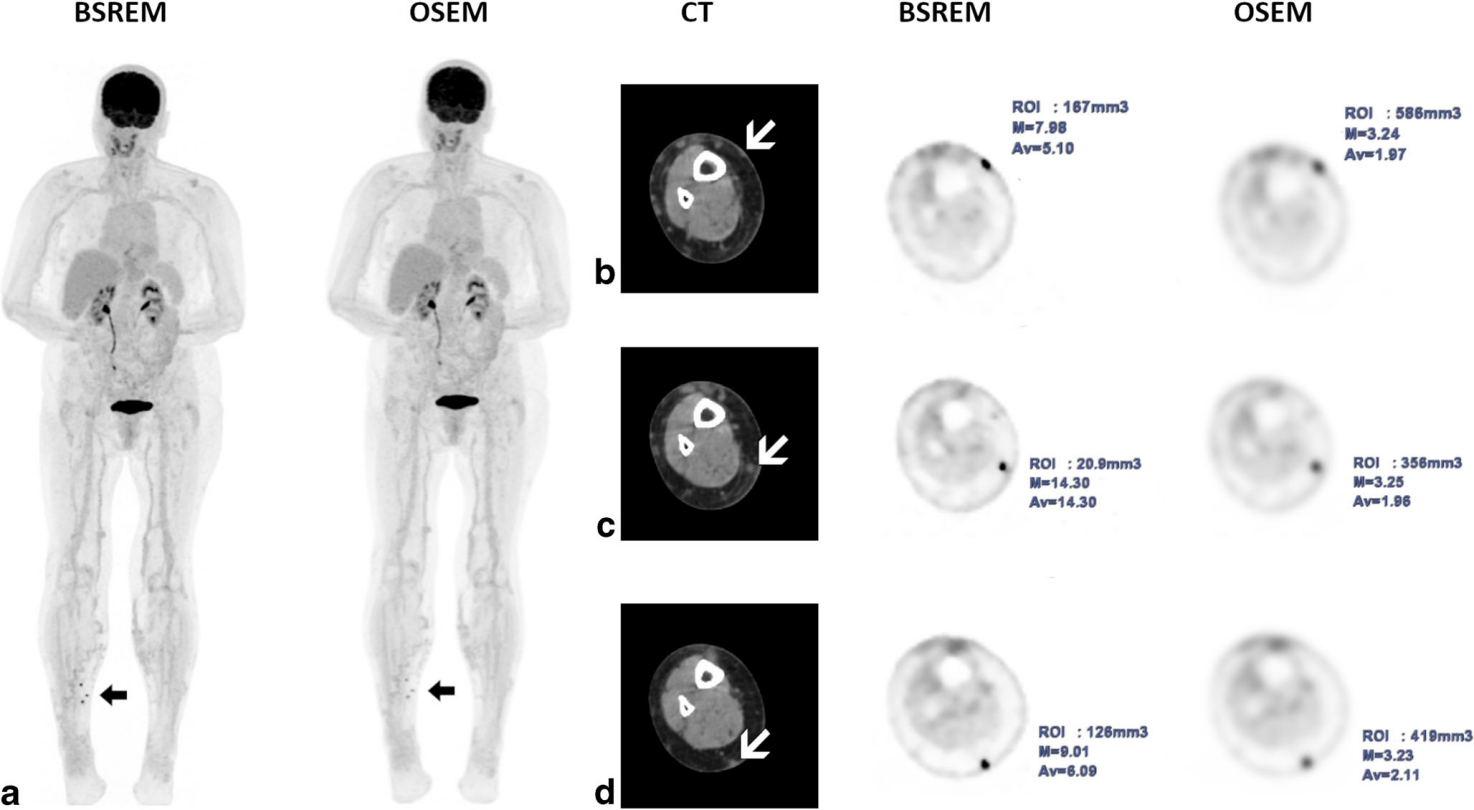
[^18^F]FDG PET/CT of a malignant melanoma patient with three right-sided lower leg in-transit metastases, visible on maximum intensity projection (MIP) images (**a**, arrow) as well as on axial CT and PET images (**b**–**d**, white arrows), better defined by BSREM reconstruction compared to OSEM reconstruction. BSREM reconstruction also yielded higher SUVmax, lower MTV, and better noise characteristics compared to OSEM reconstruction, as indicated by axial PET images (**b**–**d**)

SUVmax of ITM in BSREM and OSEM according to the anatomical location are given in Fig. 2. The highest differences in SUVmax between BSREM and OSEM were found in the head/neck and in the legs, as shown in Table 3. One representative example is given in Fig. 3.

**Table 3:** Characteristics of in-transit metastasis detected by OSEM and BSREM, according to location

In-transit metastasis (ITM) detected	OSEM	BSREM	Parameter difference in BREM vs. OSEM (%)	*p* value*
Head and neck, *n* (%)	6	19 (+ 13)	+ 216.66%	**-**
SUVmax, mean ± SD; min-max	3.41 ± 2.03 (1.26–7.93)	6.03 ± 3.35 (1.83–14.05)	+76.83%	< 0.001
TBR, mean ± SD; min-max	2.21 ± 1.45 (0.70–5.31)	3.91 ± 2.43 (1.16–11.06)	+76.92%	< 0.001
MTV (cm^3^), mean ± SD; min-max	3.21 ± 2.74 (0.61–9.14)	1.14 ± 1.22 (0.08–4.46)	− 64.48%	< 0.001
Torso, n (%)	34	47 (+ 13)	+ 38.23%	**-**
SUVmax, mean ± SD; min–max	4.56 ± 3.44 (0.68–18.57)	6.98 ± 6.32 (0.84–38.33)	+ 53.07%	< 0.001
TBR, mean ± SD; min–max	2.63 ± 1.94 (0.40–9.83)	4.01 ± 3.47 (0.49–20.39)	+52.47%	< 0.001
MTV (cm^3^), mean ± SD; min–max	1.63 ± 1.28 (0.31–5.86)	0.91 ± 3.44 (0.68–18–57)	− 44.17%	< 0.001
Arms, *n* (%)	17	23 (+ 6)	+ 35.29%	**-**
SUVmax, mean ± SD; min–max	2.97 ± 1.57 (1.10–7.37)	4.41 ± 2.94 (1.28–14.62)	+48.48%	< 0.001
TBR, mean ± SD; min–max	1.96 ± 1.07 (0.63–5.01)	2.90 ± 1.98 (0.72–10.01)	+47.95%	< 0.001
MTV (cm^3^), mean ± SD; min–max	2.67 ± 2.28 (0.44–8.70)	1.69 ± 1.80 (0.10–6.17)	− 36.70%	< 0.001
Legs, *n* (%)	149	198 (+ 48)	+ 32.21%	**-**
SUVmax, mean ± SD; min–max	5.17 ± 4.82 (0.90–46.32)	9.46 ± 8.00 (1.28–71.60)	+82.97%	< 0.001
TBR, mean ± SD; min–max	2.84 ± 2.53 (0.45–24.90)	5.29 ± 4.58 (0.71–38.29)	+86.26%	< 0.001
MTV (cm^3^), mean ± SD; min–max	1.90 ± 1.94 (0.18–10.01)	0.94 ± 1.36 (0.02–8.39)	− 50.52%	< 0.001

*Note*: *ITM*, in-transit metastasis; *TBR*, target-to-background ratio

*p value was calculated with the Wilcoxon signed-rank test

We found significant differences in PET parameters between subjects with low and high BMI (cut-off 25), both for OSEM and BSREM (*p* < 0.001), as shown in supplemental table S2. Moreover, we found a negative correlation between BMI and ITM SUVmax and TBR, and a positive correlation between BMI and blood pool SUVmean and ITM MTV, more pronounced on BSREM than on OSEM (supplemental table S3).

## Discussion

Our study is the first one (to the best of our knowledge) reporting improved detection of in-transit metastases with BSREM compared to the clinical gold standard OSEM. The major findings of our study are as follows: (1) BSREM leads to the detection of more ITM than OSEM (+39% more); (2) all ITM PET parameters (SUVmax, TBR, MTV) are significantly different between BSREM and OSEM (*p* < 0.001), with an SUVmax increase by 76.5%, a TBR increase by 77.7%, and a MTV decrease by 49.5% from OSEM to BSREM.

Several studies have highlighted the role of [^18^F]FDG PET/CT in patients with advanced-stage melanoma, especially for the detection of distant metastases during follow-up (sensitivity 82–100% and specificity 45–100%), leading to treatment change in 13–74% of stage III/IV patients [32]. As recently highlighted by Laudicella et al [33], digital PET systems and new reconstruction algorithms lead to a more accurate diagnosis, staging, and therapeutic evaluation of melanoma patients through better image quality, higher spatial resolution, and more accurate image reconstruction. Aljared et al [34] reported added value of BSREM reconstruction in a melanoma patient, where four [^18^F]FDG-avid ITMs were detected only on BSREM reconstruction. It was assumed that BSREM may have implications for the detection of small lesions [35], such as ITM, and might influence therapeutic decisions.

Our study’s results validate this hypothesis: BSREM identified 81 ITM more than OSEM (287 versus 206; +39%). In 20 patients, the presence of ITM was detected only with BSREM, but missed with OSEM. This can be attributed to the fact that focal uptake with higher activity concentration tends to converge faster compared to the uptake with lower activity concentration, so the convergence benefit of BSREM reconstruction in providing more convergent foci compared to OSEM [20, 23].

**Fig. 4:**
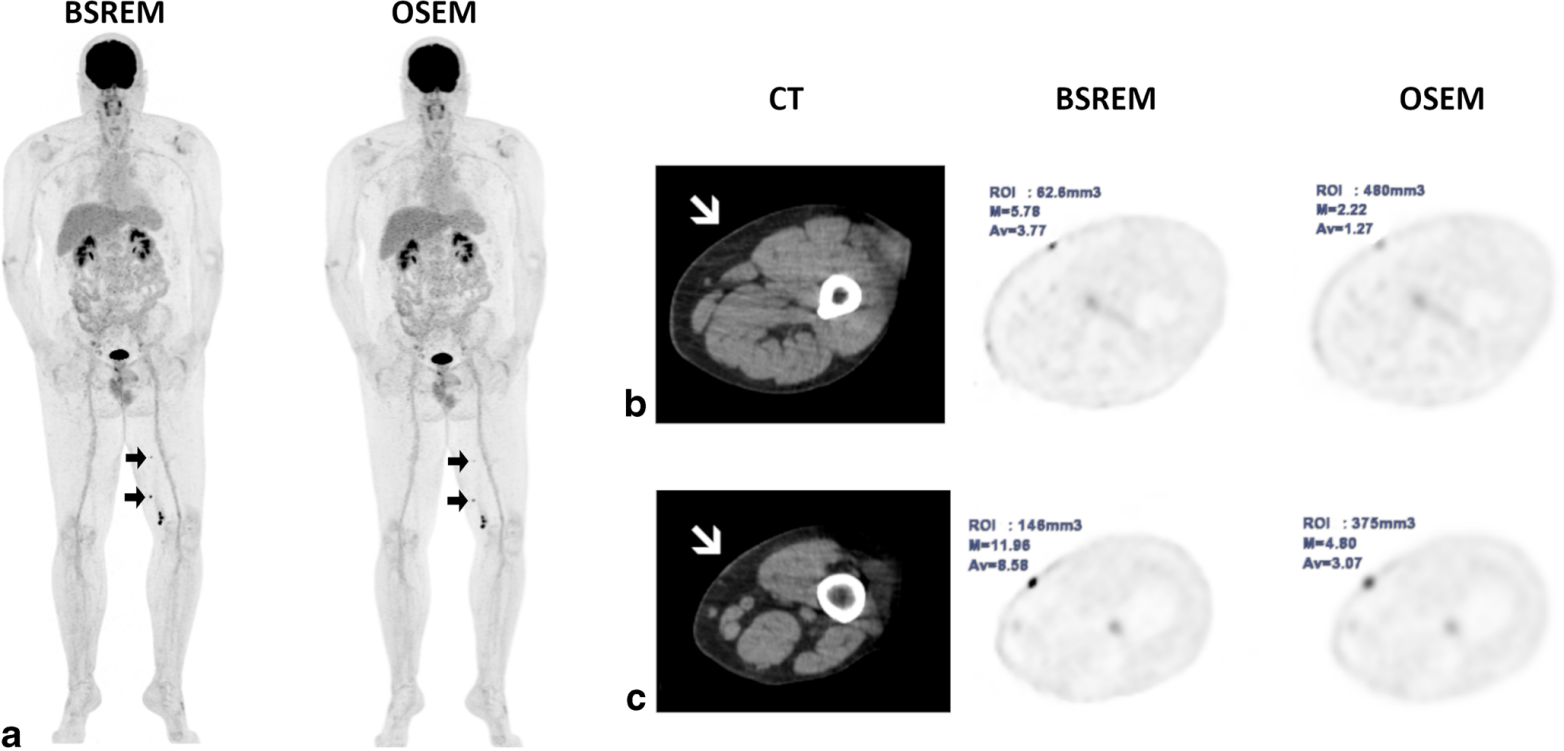
[^18^F]FDG PET/CT of a malignant melanoma patient with several left-sided leg in-transit metastases, visible on MIP images (**a**). Of these, the two proximal ones (black arrows) were detected only at BSREM reconstruction. As shown in the axial images (**b** and **c**), BSREM yielded higher SUVmax, lower MTV, and better noise characteristics compared to OSEM. Despite their high uptake at BSREM and the location highly suspicious for ITM, these two lesions had no anatomical correlate on CT (white arrows), except for a slight skin thickening at one site. Both lesions were subsequently confirmed by histopathology

Thereof, a single lesion was detected in 17 cases and 2 to 5 lesions were detected in 3 cases. BSREM lead to the identification of 1 lesion with an uptake below blood pool background (SUVmax 0.84 and TBR 0.48), 18 lesions with MTV < 0.1 cm^3^, and 2 lesions with MTV = 0.02 cm^3^, corresponding to a diameter of approximately 3 mm, which is in line with Baratto et al [36], who detected lesions < 0.7 cm with digital PET systems (Fig. 4). Moreover, MTV was increasing particularly with OSEM in lesions with low SUVmax, translating into the observed blurring effect with OSEM, and such may serve as a measure of poor detectability. This also implies that MTV is overestimated using OSEM, particularly in small lesions.

Patients presenting with ITM on clinical examination should undergo restaging including physical examination and whole-body imaging in order to guide therapeutic options [12]. [^18^F]FDG PET/CT allows both ITM detection and whole-body restaging at the same time. Moreover, the identification of the exact number and site of in-transit metastases is fundamental for the choice of the optimal therapy: ITMs are typically resected if less than 3–4 lesions and none larger than 5 cm; otherwise, locoregional treatment should be evaluated, with a preference for TVEC in the torso or head/neck ITM. Systemic therapy should be chosen with a concurrent clinically evident metastatic or nodal disease with or without the aforementioned simultaneous specific ITM treatment [16, 35]. In these terms, the positive impact that BSREM, comparing to OSEM reconstruction, could have in the evaluation of [^18^F]FDG PET/CT images is evident.

Obviously, future prospective and comparative studies with other reference methods for ITM detention are needed to determine the added value of [^18^F]FDG PET/CT in evaluating ITMs in a clinical setting and to analyze possible associations between ITM number and site with recurrence-free survival (RFS), distant metastasis-free survival (DMFS), and melanoma-specific survival. In 2014, Solivetti et al [37] studied whether US could be replaced or integrated with other techniques, such as [^18^F]FDG PET/CT and telethermography (TT). All 52 ITMs in 15 patients in their study were detected by HF-US (100%), 24/52 were detected by PET/CT (42.6%), and 15/52 were detected by TT (27.7%). PET/CT reported 3.7% false positives, while no false positives were reported by TT. Our study did not aim to compare different examination techniques; however, we hypothesize that these results may be different in a larger cohort of patients and with the use of new digital PET/CT systems. In our cohort, only 8 out of 287 lesions finally resulted to be false positive (0.96% of cases).

Finally, the impact of [^18^F]FDG PET/CT should be evaluated considering also the contextual whole-body (re)staging, which is compulsory after ITM detection. Two different retrospective studies on two large cohorts of melanoma patients with ITM (380 German and 11614 Australian patients, respectively) found that lymph node involvement is an important prognostic factor in this cohort [38, 39]. Even if it was not the aim of our study, we reported similar results with a higher favorable outcome in patients with only ITM (34/54) compared to patients with ITM and lymph node metastases (10/14) and patients with ITM and distant metastases (15/32). Also in this context, we expect that further studies will assess the benefit of BSREM reconstruction in the evaluation of the global tumor burden in malignant melanoma. Such would allow for a more accurate assessment of the state of the disease, taking into account the whole-body tumor burden, and its impact on staging and follow-up.

Our study is not exempt from limitations. First, although readers were blinded to the type of reconstruction used, an experienced reader may recognize the actual algorithm used based on the reconstructed images. Second, not all ITMs were proven by histology. However, all lesions were located in the subcutaneous adipose tissue between the primary tumor site and the regional nodal basin, which is suggestive for ITM, and lesions were also suspected to represent ITM on ultrasound. It is known that rarely also ectopic lymph nodes may exist in the subcutaneous adipose tissue. Hence, some of the “ITMs” could actually have represented lymph node metastases, which in turn even would have led to an upstaging of patients. Notably, lesions suspicious for ITM were not detected in anatomical regions other than the one that harbored the primary tumor in our cohort. Since PET/CT intrinsically represents a standard of reference for ITM detection, we cannot comment on false-negative lesions. However, reporting diagnostic accuracy of PET was also not the thrust of our study, and such would require a comparison with ultrasound in order to make sense. Of note, in our cohort, no additional ITMs were detected with ultrasound besides the ones detected with PET. Third, the exclusion of melanoma patients scanned with analog PET/CT may have reduced the possibility to give epidemiology information about ITM frequency related to the entire patient population with malignant melanoma.

## Conclusion

The detection of in-transit metastases in [^18^F]FDG PET/CT has significantly impacted by the use of BSREM reconstruction. BSREM detects significantly more (+39%) in-transit metastases than OSEM, with a significant difference (all *p* < 0.001) in ITM SUVmax (+76.5%), TBR (+77.7%), and MTV (- 49.5%) compared to OSEM.

## Supplementary information

ESM 1 (DOCX 1424 kb)

## Abbreviations

[^18^F]FDG: 2-Deoxy-2-[18F]fluoro-D-glucose
AJCC: American Joint Committee on Cancer
BMI: Body mass index
BPL: Bayesian penalized likelihood
BRAF: v-Raf murine sarcoma viral oncogene homolog B
BSREM: Block sequential regularized expectation maximization
CECT: Contrast-enhanced computed tomography
CMM: Cutaneous malignant melanoma
CT: Computed tomography
ITM: In-transit metastases
MEK: Mitogen-activated protein kinase
MIP: Maximum intensity projection
MR: Magnetic resonance
OSEM: Ordered subset expectation maximization
PET: Positron emission tomography
PSF: Point spread function
SiPM: Silicon photomultiplier
SUVmax: Maximum standardized uptake value
TOF: Time of flight
TVEC: Talimogene laherparepvec
VOI: Volume of interest
