# Supplementary material for: Improved detection of in-transit metastases of malignant melanoma with BSREM reconstruction in digital [18F]FDG PET/CT
Source: Eur Radiol. 2021 Mar 25;31(10):8011–20. doi: 10.1007/s00330-021-07852-7 (PMC8452544; doi:10.1007/s00330-021-07852-7)
Supplement: Supplementary file 1 — (DOCX 1424 kb) [file 330_2021_7852_MOESM1_ESM.docx]

**SUPPLEMENT MATERIAL**

**Table S1**. Malignant melanoma TNM classification and stage according to the American Joint Committee on Cancer (AJCC) 8^th^ edition guidelines.

|  | **Before PET/CT scan** | **After PET/CT scan** | **At last follow-up** |
| --- | --- | --- | --- |
| *T classification*, n (%)  *T1*  *T2*  *T3*  *T4*  *Tx* | 4 (4.0)  29 (29.0)  31 (31.0)  25 (25.0)  11 (11.0) | 4 (4.0)  29 (29.0)  31 (31.0)  26 (26.0)  10 (10.0) | 4 (4.0)  29 (29.0)  31 (31.0)  26 (26.0)  10 (10.0) |
| *N classification*, n (%)  *N0*  *N1*  *N2*  *N3*  *Nx* | 12 (12.0)  17 (17.0)  34 (34.0)  28 (28.0)  9 (9.0) | 9 (9.0)  17 (17.0)  32 (32.0)  39 (39.0)  3 (3.0) | 9 (9.0)  17 (17.0)  31 (31.0)  41 (41.0)  2 (2.0) |
| *In-transit metastasis*, n (%)  *Yes*  *No* | 49 (49.0)  51 (51.0) | 75 (75.0)  25 (25.0) | 76 (76.0)  24 (24.0) |
| *M classification*, n (%)  *M0*  *M1a*  *M1b*  *M1c*  *M1d*  *Mx* | 65 (65.0)  10 (10.0)  2 (2.0)  10 (10.0)  5 (5.0)  8 (8.0) | 61 (61.0)  16 (16.0)  5 (5.0)  12 (12.0)  6 (6.0)  0 (0.0) | 52 (52.0)  13 (13.0)  9 (9.0)  16 (16.0)  10 (10.0)  0 (0.0) |
| *Stage*, n (%)  *0*  *IA*  *IB*  *IIA*  *IIB*  *IIC*  *IIIA*  *IIIB*  *IIIC*  *IIID*  *IV* | -  -  6 (6.0)  -  5 (5.0)  -  4 (4.0)  9 (9.0)  30 (30.0)  19 (19.0)  27 (27.0) | -  -  -  -  2 (2.0)  -  -  5 (5.0)  29 (29.0)  24 (24.0)  40 (40.0) | -  -  -  -  1 (1.0)  -  -  5 (5.0)  26 (26.0)  20 (20.0)  48 (48.0) |
| *Gershenwald JE, Scolyer RA (2018) Melanoma Staging: American Joint Committee on Cancer (AJCC) 8th Edition and Beyond. Ann. Surg. Oncol. 25:2105–2110* | | | |

**Table S2**. Differences in PET parameters (expressed as *p*-values) between OSEM and BSREM reconstructions, stratified by gender, BMI and age for the entire cohort and for the sub-group of ITM detected only with BSREM reconstruction (and retrospectively analysed with OSEM reconstruction).

| **ITM entire cohort** | **OSEM** | | | | **BSREM** | | | |
| --- | --- | --- | --- | --- | --- | --- | --- | --- |
|  | **Blood pool SUVmean** | **ITM SUVmax** | **ITM TBR** | **ITM MTV (cm3)** | **Blood pool SUVmean** | **ITM SUVmax** | **ITM TBR** | **ITM MTV (cm3)** |
| *Gender* | <0.001 | 0.286 | 0.714 | 0.275 | <0.001 | 0.260 | 0.868 | 0.010 |
| *BMI*  *(cut-off 30)* | <0.001 | 0.862 | 0.349 | 0.067 | <0.001 | 0.298 | 0.036 | 0.007 |
| *BMI*  *(cut-off 25)* | <0.001 | 0.456 | 0.771 | <0.001 | <0.001 | 0.442 | 0.098 | <0.001 |
| *Age*  *(cut-off 45)* | 0.070 | 0.112 | 0.253 | 0.281 | 0.151 | 0.051 | 0.144 | 0.410 |
|  | | | | | | | | |
| **ITM detected only by BSREM** | **OSEM** | | | | **BSREM** | | | |
|  | **Blood pool SUVmean** | **ITM SUVmax** | **ITM TBR** | **ITM MTV (cm3)** | **Blood pool SUVmean** | **ITM SUVmax** | **ITM TBR** | **ITM MTV (cm3)** |
| *Gender* | <0.001 | 0.221 | 0.041 | 0.025 | <0.001 | 0.708 | 0.232 | 0.423 |
| *BMI*  *(cut-off 30)* | 0.035 | 0.631 | 0.755 | 0.457 | 0.015 | 0.280 | 0.097 | 0.033 |
| *BMI*  *(cut-off 25)* | 0.010 | 0.260 | 0.973 | 0.052 | 0.008 | 0.978 | 0.469 | 0.005 |
| *Age*  *(cut-off 45)* | 0.271 | 0.429 | 0.666 | 0.238 | 0.195 | 0.096 | 0.158 | 0.322 |
| **Note**: BMI = body mass index, ITM = in-transit metastasis, TBR = target to background ratio  **p-value* was calculated with Mann Whitney U test | | | | | | | | |

**Table S3**. Pearson correlation of BMI, age and PET parameters (SUVmean of mediastinal blood pool; SUVmax, TBR and MTV of ITM) in OSEM and BSREM reconstructions, respectively, for the entire cohort and for the sub-group of ITM detected only with BSREM reconstruction (and retrospectively analysed with OSEM reconstruction).

| **ITM entire cohort** | **OSEM** | | | | **BSREM** | | | |
| --- | --- | --- | --- | --- | --- | --- | --- | --- |
|  | **Blood pool SUVmean** | **ITM SUVmax** | **ITM TBR** | **ITM MTV**  **(cm3)** | **Blood pool SUVmean** | **ITM SUVmax** | **ITM TBR** | **ITM MTV**  **(cm3)** |
| *BMI*  *Correlation coefficient*  *p- value* | **  0.399  **<0.001** | -0.008  0.896 | -0.07  0.237 | 0.130  **0.028** | **  0.401  **<0.001** | -0.120  **0.041** | -0.185  **0.002** | 0.083  0.163 |
| *Age*  *Correlation coefficient*  *p-value* | 0.197  **<0.001** | 0.09  0.13 | 0.067  0.261 | 0.063  0.287 | 0.194  **<0.001** | 0.095  0.107 | 0.071  0.231 | 0.120  **0.043** |
|  | | | | | | | | |
| **ITM detected only by BSREM** | **OSEM** | | | | **BSREM** | | | |
|  | **Blood pool SUVmean** | **ITM SUVmax** | **ITM TBR** | **ITM MTV**  **(cm3)** | **Blood pool SUVmean** | **ITM SUVmax** | **ITM TBR** | **ITM MTV**  **(cm3)** |
| *BMI*  *Correlation coefficient*  *p-value* | 0.295  **0.007** | 0.026  0.815 | -0.74  0.510 | 0.108  0.335 | 0.299  **0.007** | -0.112  0.322 | -0.184  0.101 | 0.229  **0.040** |
| *Age*  *Correlation coefficient*  *p-value* | 0.196  0.079 | 0.116  0.304 | 0.037  0.745 | -0.135  0.231 | 0.185  0.098 | 0.182  0.103 | 0.120  0.284 | -0.097  0.388 |
| **Note**: BMI = body mass index, ITM = in-transit metastasis, TBR = target-to-background ratio  *correlation and *p-value* were calculated with Pearson test  **only variables with a correlation > ±0.35 and a *p-value* < 0.05 were considered significantly correlated | | | | | | | | |
